# The complete mitochondrial genomes of two skipper genera (Lepidoptera: Hesperiidae) and their associated phylogenetic analysis

**DOI:** 10.1038/s41598-018-34107-1

**Published:** 2018-10-25

**Authors:** Yuke Han, Zhenfu Huang, Jing Tang, Hideyuki Chiba, Xiaoling Fan

**Affiliations:** 1 Department of Entomology, College of Agriculture, South China Agricultural University, Guangzhou, 510642 China; 2 B. P. Bishop Museum, Honolulu, Hawaii 96817-0916 USA

## Abstract

The systematic positions of two hesperiid genera, *Apostictopterus* and *Barca* (Lepidoptera: Hesperiidae), remain ambiguous. We sequenced and annotated the two mitogenomes of *Apostictopterus fuliginosus* and *Barca bicolor* and inferred the phylogenetic positions of the two genera within the Hesperiidae based on the available mitogenomes. The lengths of the two circular mitogenomes of *A. fuliginosus* and *B. bicolor* are 15,417 and 15,574 base pairs (bp), respectively. These two mitogenomes show similar AT skew, GC skew, codon usage and nucleotide bias of AT: the GC skew of the two species is negative, and the AT skew of *A. fuliginosus* is negative, while the AT skew of *B. bicolor* is slightly positive. The largest intergenic spacer is located at the same position between *trnQ* and *ND2* in *A. fuliginosus* (73 bp) and *B. bicolor* (72 bp). Thirteen protein-coding genes (PCGs) start with ATN codons except for *COI*, which starts with CGA. The control regions of both mitogenomes possess a long tandem repeat, which is 30 bp long in *A. fuliginosus*, and 18 bp in *B. bicolor*. Bayesian inference and maximum likelihood methods were employed to infer the phylogenetic relationships, which suggested that *A. fuliginosus* and *B. bicolor* belong in the subfamily Hesperiinae.

## Introduction

Skipper butterflies (Lepidoptera: Hesperiidae) include approximately 4,000 species in 567 genera worldwide^1^ and account for a fifth of the world’s butterfly fauna^2^. Despite considerable efforts in recent years^3–5^, the higher-level phylogenetic relationships within the family Hesperiidae are still unsatisfactorily resolved. The taxonomic affinities of many genera are not conclusive, even at the subfamily level^6^, including *Apostictopterus* and *Barca*.

The taxonomic positions of the two monotypic genera *Apostictopterus* and *Barca* have been controversial. They were assigned to the *Heteropterus* group of the subfamily Hesperiinae close to the *Astictopterus* group in Evans’s classification^7^, while Chou^8^ assigned *Apostictopterus* to the tribe Astictopterini and *Barca* to the tribe Heteropterini. Since Higgins^9^, the *Heteropterus* group of Evans has widely been regarded as Heteropterinae at the subfamily level. In previous studies^1,10^, these two genera were both treated as members of the subfamily Heteropterinae. However, on the basis of morphological evidence, Warren *et al*.^6^ were more likely to place them in Hesperiinae.

The difficulty of morphologically based phylogenetic systematics has been shown, whereas molecular phylogeny has been contributing to the development of a more stable classification. Since mitochondria are characterised by maternal inheritance, a rapid evolutionary rate, and little or no genetic recombination, they have been extensively used in the field of genetics and evolutionary biology^11–14^. Insect mitochondrial genomes (mitogenomes) are typically compact circular molecules of 15–18 kb containing 37 genes, including 13 protein-coding genes (PCGs), 22 transfer RNAs (tRNAs), and two ribosomal RNAs (rRNAs)^15,16^. In addition, the mitogenome mostly contains a control region (an AT-rich region due to a high A + T content) that has a longer sequence than the other regions and embraces essential regulatory elements for transcription and replication^16–20^. However, this region cannot be well sequenced by high-throughput sequencing techniques, as the depth of coverage is strongly positively correlated with the GC content^21^.

Mitogenomes are data rich and relatively accessible source of information. Condamine^21^ had obtained promising results on the genus-level relationships of swallowtail butterflies using mitogenomes. Thus far, 30 complete or nearly complete mitogenomes of skippers have been sequenced. In this study, we sequenced two additional complete mitogenomes of *A. fuliginosus* and *B. bicolor* and then elucidated the composition of the genomes. Finally, we inferred the phylogenetic relationships from the 27 available mitogenomes within the Hesperiidae^4,5,22–26^. We did not use three mitogenomes. *Polytremis jigongi* and *Polytremis nascens* showed very low homology to the other species. There are two mitogenomes of *Daimio tethys* that are basically in line, so we randomly selected the one from Korea based on a computation-efficient strategy.

## Results and discussion

### Genome structure and organization

**Figure 1:**
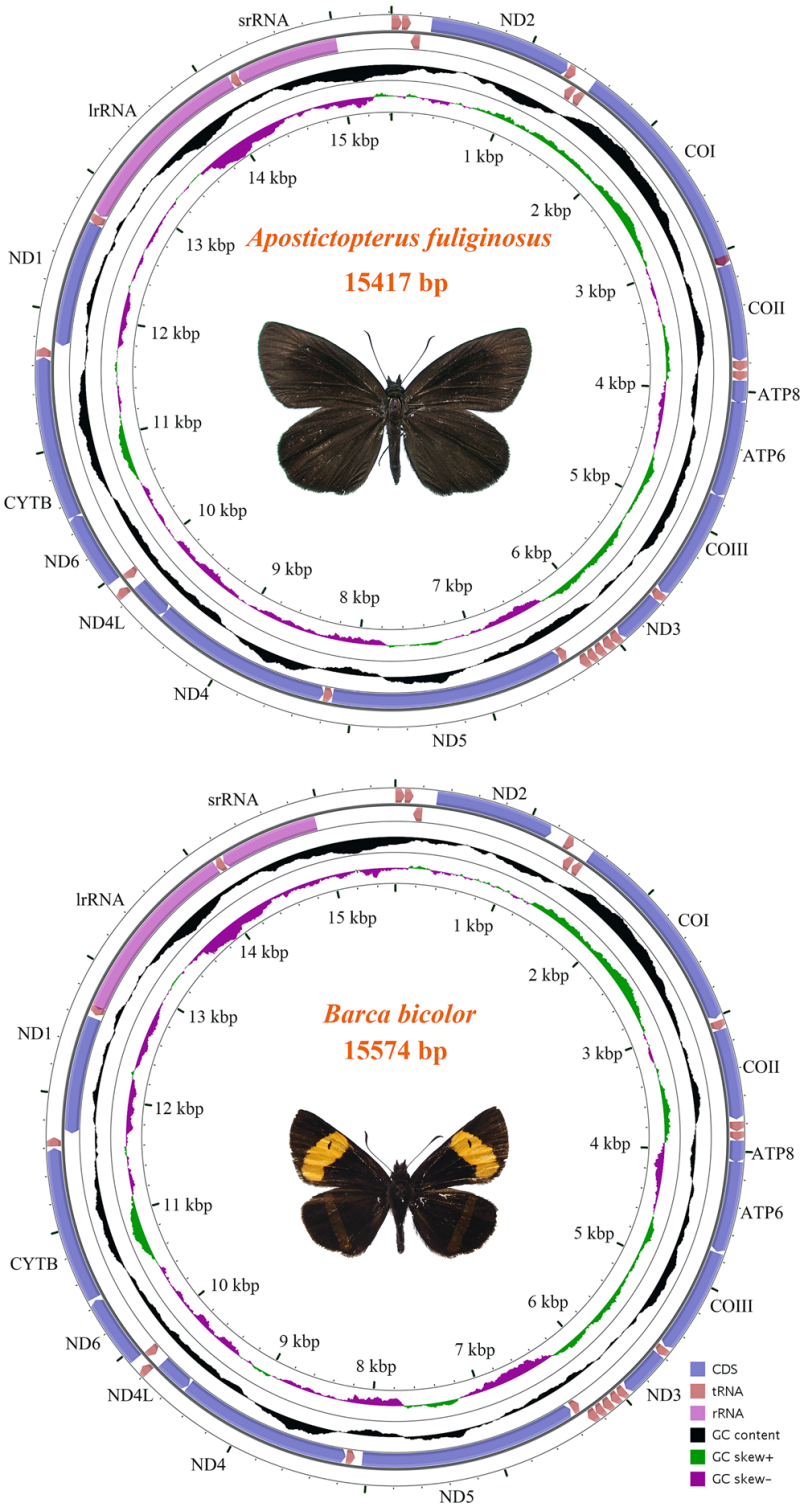
Circular map of the mitogenomes of *A. fuliginosus* and *B. bicolor*.

The complete mitogenomes of *A. fuliginosus* and *B. bicolor* are 15,417 bp and 15,574 bp (Fig. 1), respectively, which are similar to other hesperiid mitogenomes (Table 1). The organisations of *A. fuliginosus* and *B. bicolor* are shown in Table 1. Similar to most typical insect mitogenomes, these two species harbours 13 protein-coding genes (*ATP6, ATP8, Cytb, COI-COIII, ND1-ND6*, and *ND4L*), 22 transfer RNAs (tRNAs), two ribosomal RNAs (rRNA: *lrRNA* and *srRNA*), and an AT-rich region. These assembly units are identical to those of the other skippers, and the encoding protein genes’ ORF direction is the same as in most skippers. Both mitogenomes have 15 intergenic regions. The maximum intervals of *A. fuliginosus* and *B. bicolor*, both between *trnQ* with *ND2*, are 73 bp and 72 bp, respectively. Only a few genes (four PCGs, eight tRNAs, and two rRNAs) are from the N strand, and the remaining 23 genes (nine PCGs and 14 tRNAs) are from the J strand. The nucleotide composition of *A. fuliginosus* is A (40.1%), T (40.6%), C (11.8%), and G (7.4%); the AT nucleotide content is as high as 80.7%. In *B. bicolor*, the composition is A (40.0%), T (39.4%), C (12.9%), and G (7.7%); the AT nucleotide content is as high as 79.4%. In these two mitogenomes, the GC skew of two mitogenomes and the AT skew of *A. fuliginosus* are negatively biased, while the AT skew of *B. bicolor* has a slightly positive bias (Supplementary Material S1).

**Table 1:** Organization of the *A. fuliginosus* and *B. bicolor* mitogenomes.

Specices	Gene	Direction	Location	Size	Anticodon	Start codon	Stop codon	Intergenic nucleotide
*Apostictopterus fuliginosus*	*tRNA* ^*Met*^	F	1–74	74	ATG			0
	*tRNA* ^*Ile*^	F	75–138	64	ATC			3
	*tRNA* ^*Gln*^	R	142–210	69	CAA			73
	*ND2*	F	284–1,297	1,014		ATT	TAA	1
	*tRNA* ^*Trp*^	F	1,299–1,363	65	TGA			−8
	*tRNA* ^*Cys*^	R	1,356–1,420	65	TGC			12
	*tRNA* ^*Tyr*^	R	1,433–1,498	66	TAC			7
	*COI*	F	1,506–3,036	1,531		CGA	T–	0
	*tRNA* ^*Leu*^	F	3,037–3,104	68	TTA			0
	*COII*	F	3,105–3,783	679		ATG	T–	0
	*tRNA* ^*Lys*^	F	3,784–3,854	71	AAG			2
	*tRNA* ^*Asp*^	F	3,857–3,923	67	GAC			0
	*ATP8*	F	3,924–4,082	159		ATT	TAA	−7
	*ATP6*	F	4,076–4,753	678		ATG	TAA	−1
	*COIII*	F	4,753–5,538	786		ATG	TAA	2
	*tRNA* ^*Gly*^	F	5,541–5,607	67	GGA			0
	*ND3*	F	5,608–5,961	354		ATT	TAA	3
	*tRNA* ^*Ala*^	F	5,965–6,032	68	GCA			−1
	*tRNA* ^*Arg*^	F	6,032–6,100	69	CGA			7
	*tRNA* ^*Asn*^	F	6,108–6,172	65	AAC			4
	*tRNA* ^*Ser*^	F	6,177–6,237	60	AGC			0
	*tRNA* ^*Glu*^	F	6,238–6,304	67	GAA			38
	*tRNA* ^*Phe*^	R	6,343–6,407	65	TTC			0
	*ND5*	R	6,408–8,148	1,741		ATT	T–	0
	*tRNA* ^*His*^	R	8,149–8,215	67	CAC			0
	*ND4*	R	8,216–9,554	1,339		ATG	T–	−1
	*ND4L*	R	9,554–9,838	285		ATG	TAA	2
	*tRNA* ^*Thr*^	F	9,841–9,905	65	ACA			0
	*tRNA* ^*Pro*^	R	9,906–9,970	65	CCA			2
	*ND6*	F	9,973–10,509	537		ATA	TAA	−1
	*Cytb*	F	10,509–11,660	1,152		ATG	TAA	3
	*tRNA* ^*Ser*^	F	11,664–11,730	67	TCA			19
	*ND1*	R	11,750–12,688	939		ATG	TAA	−6
	*tRNA* ^*Leu*^	R	12,683–12,757	75	CTA			0
	*lrRNA*	R	12,758–14,172	1,415				0
	*tRNA* ^*Val*^	R	14,173–14,237	65	GTA			−1
	*srRNA*	R	14,237–15,010	774				−1
	AT-rich region		15,011–15,417	407				0
*Barca bicolor*	*tRNA* ^*Met*^	F	1–68	68	ATG			0
	*tRNA* ^*Ile*^	F	69–132	64	ATC			3
	*tRNA* ^*Gln*^	R	136–204	69	CAA			72
	*ND2*	F	277–1,290	1,014		ATT	TAA	4
	*tRNA* ^*Trp*^	F	1,295–1,359	65	TGA			8
	*tRNA* ^*Cys*^	R	1,352–1,419	68	TGC			13
	*tRNA* ^*Tyr*^	R	1,433–1,497	65	TAC			7
	*COI*	F	1,505–3,035	1,531		CGA	T–	0
	*tRNA* ^*Leu*^	F	3,036–3,103	68	TTA			0
	*COII*	F	3,104–3,782	679		ATG	T–	0
	*tRNA* ^*Lys*^	F	3,783–3,853	71	AAG			1
	*tRNA* ^*Asp*^	F	3,855–3,920	66	GAC			0
	*ATP8*	F	3,921–4,082	162		ATC	TAA	−7
	*ATP6*	F	4,076–4,753	678		ATG	TAA	−1
	*COIII*	F	4,753–5,538	786		ATG	TAA	2
	*tRNA* ^*Gly*^	F	5,541–5,606	66	GGA			0
	*ND3*	F	5,607–5,960	354		ATT	TAA	3
	*tRNA* ^*Ala*^	F	5,964–6,026	63	GCA			0
	*tRNA* ^*Arg*^	F	6,027–6,093	67	CGA			3
	*tRNA* ^*Asn*^	F	6,097–6,163	67	AAC			4
	*tRNA* ^*Ser*^	F	6,168–6,229	62	AGC			0
	*tRNA* ^*Glu*^	F	6,230–6298	69	GAA			−2
	*tRNA* ^*Phe*^	R	6,297–6,360	64	TTC			0
	*ND5*	R	6,361–8,098	1,738		ATT	T–	0
	*tRNA* ^*His*^	R	8,099–8,164	66	CAC			−1
	*ND4*	R	8,164–9,504	1,341		ATG	TAA	−1
	*ND4L*	R	9,504–9,788	285		ATG	TAA	2
	*tRNA* ^*Thr*^	F	9,791–9,855	65	ACA			0
	*tRNA* ^*Pro*^	R	9,856–9,921	66	CCA			2
	*ND6*	F	9,924–10,460	537		ATA	TAA	−1
	*Cytb*	F	10,460–11,611	1,152		ATG	TAA	1
	*tRNA* ^*Ser*^	F	11,613–11,678	66	TCA			18
	*ND1*	R	11,697–12,635	939		ATG	TAA	−6
	*tRNA* ^*Leu*^	R	12,630–12,703	74	CTA			0
	*lrRNA*	R	12,704–14,122	1,419				0
	*tRNA* ^*Val*^	R	14,123–14,187	65	GTA			0
	*srRNA*	R	14,188–14,960	773				0
	AT-rich region		14,961–15,574	614				0

In the column intergenic length, the positive number indicates interval base pairs between genes, while the negative number indicates the overlapping base pairs between genes.

### Protein-coding genes (PCGs)

**Figure 2:**
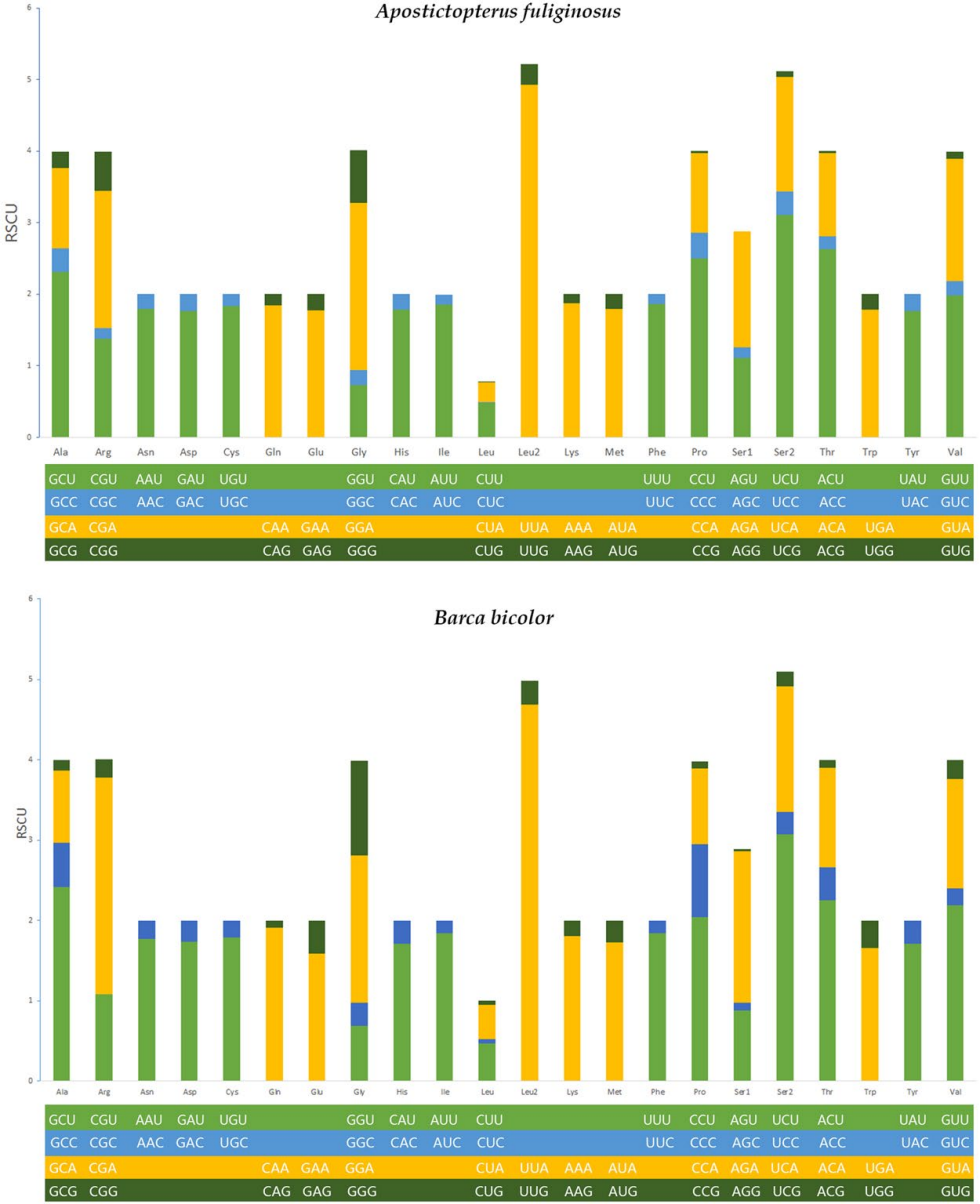
The relative synonymous codon usage (RSCU) in the mt-genomes of *A. fuliginosus* and *B.* *bicolor*.

The PCGs of the two mitogenomes encode a total of 3,730 (*A. fuliginosus*) and 3,731 (*B. bicolor*) amino acids, which account for 72.6% and 71.9% of *A. fuliginosus* and *B. bicolor*, respectively. All PCGs in both mitogenomes start with typical ATN codons, except for *COI*, which is initiated by CGA, as is common in Lepidoptera. Stop codons in the PCGs include two types: TAA or T. Though incomplete stop codons always appear in lepidopteran mitogenomic PCGs, translation will not be affected at all because the codons will be automatically filled by added As during the transcription process^27^. We calculated the relative synonymous codon usage (RSCU) of the PCGs in the two mitogenomes (Table 2). According to the RSCU analyses, TTT (F), ATT (I), TTA (L) and ATA (M) were the four most frequently used codons. In both species, leucine, isoleucine, phenylalanine and serine are the most frequent PCG amino acids (Fig. 2).

**Table 2:** Codon number and RSCU in the *A*. *fuliginosus* and *B. bicolor* mitochondrial PCGs.

Specices	Codon	Count	RSCU	Codon	Count	RSCU	Codon	Count	RSCU	Codon	Count	RSCU
*Apostictopterus fuliginosus*	**UUU(F)**	339	1.87	UCU(S)	123	3.11	UAU(Y)	165	1.77	UGU(C)	34	1.84
	**UUC(F)**	23	0.13	UCC(S)	13	0.33	UAC(Y)	21	0.23	UGC(C)	3	0.16
	**UUA(L)**	462	4.93	UCA(S)	63	1.6	UAA(*)	0	0	UGA(W)	85	1.79
	**UUG(L)**	27	0.29	UCG(S)	3	0.08	UAG(*)	0	0	UGG(W)	10	0.21
	**CUU(L)**	45	0.48	CCU(P)	76	2.5	CAU(H)	60	1.79	CGU(R)	18	1.38
	**CUC(L)**	1	0.01	CCC(P)	11	0.36	CAC(H)	7	0.21	CGC(R)	2	0.15
	**CUA(L)**	26	0.28	CCA(P)	34	1.11	CAA(Q)	63	1.85	CGA(R)	25	1.92
	**CUG(L)**	1	0.01	CCG(P)	1	0.03	CAG(Q)	5	0.15	CGG(R)	7	0.54
	**AUU(I)**	445	1.86	ACU(T)	104	2.63	AAU(N)	224	1.8	AGU(S)	44	1.11
	**AUC(I)**	33	0.13	ACC(T)	7	0.18	AAC(N)	24	0.2	AGC(S)	6	0.15
	**AUA(M)**	259	1.8	ACA(T)	46	1.16	AAA(K)	109	1.88	AGA(S)	64	1.62
	**AUG(M)**	30	0.2	ACG(T)	1	0.03	AAG(K)	7	0.12	AGG(S)	0	0
	**GUU(V)**	59	1.98	GCU(A)	70	2.31	GAU(D)	53	1.77	GGU(G)	35	0.73
	**GUC(V)**	6	0.2	GCC(A)	10	0.33	GAC(D)	7	0.23	GGC(G)	10	0.21
	**GUA(V)**	51	1.71	GCA(A)	34	1.12	GAA(E)	64	1.78	GGA(G)	113	2.34
	**GUG(V)**	3	0.1	GCG(A)	7	0.23	GAG(E)	8	0.22	GGG(G)	35	0.73
*Barca bicolor*	**UUU(F)**	332	1.84	UCU(S)	119	3.07	UAU(Y)	163	1.71	UGU(C)	35	1.79
	**UUC(F)**	28	0.16	UCC(S)	11	0.28	UAC(Y)	28	0.29	UGC(C)	4	0.21
	**UUA(L)**	445	4.69	UCA(S)	61	1.57	UAA(*)	0	0	UGA(W)	78	1.66
	**UUG(L)**	28	0.3	UCG(S)	7	0.18	UAG(*)	0	0	UGG(W)	16	0.34
	**CUU(L)**	45	0.47	CCU(P)	65	2.04	CAU(H)	58	1.71	CGU(R)	14	1.08
	**CUC(L)**	5	0.05	CCC(P)	29	0.91	CAC(H)	10	0.29	CGC(R)	0	0
	**CUA(L)**	41	0.43	CCA(P)	30	0.94	CAA(Q)	66	1.91	CGA(R)	35	2.7
	**CUG(L)**	5	0.05	CCG(P)	3	0.09	CAG(Q)	3	0.09	CGG(R)	3	0.23
	**AUU(I)**	417	1.84	ACU(T)	87	2.25	AAU(N)	216	1.77	AGU(S)	34	0.88
	**AUC(I)**	37	0.16	ACC(T)	16	0.41	AAC(N)	28	0.23	AGC(S)	4	0.1
	**AUA(M)**	251	1.73	ACA(T)	48	1.24	AAA(K)	103	1.81	AGA(S)	73	1.88
	**AUG(M)**	40	0.27	ACG(T)	4	0.1	AAG(K)	11	0.19	AGG(S)	1	0.03
	**GUU(V)**	74	2.19	GCU(A)	75	2.42	GAU(D)	53	1.74	GGU(G)	33	0.69
	**GUC(V)**	7	0.21	GCC(A)	17	0.55	GAC(D)	8	0.26	GGC(G)	14	0.29
	**GUA(V)**	46	1.36	GCA(A)	28	0.9	GAA(E)	59	1.59	GGA(G)	87	1.83
	**GUG(V)**	8	0.24	GCG(A)	4	0.13	GAG(E)	15	0.41	GGG(G)	56	1.18

### Ribosomal RNA and Transfer RNA genes

The two rRNA genes (*lrRNA*, *srRNA*) encoding the small and large ribosomal subunits are located between *trnL*^*(CUN)*^ and *trnV* and between *trnV* and the AT-rich region. The *lrRNA* and *srRNA* lengths are 1,415 and 774 bp, respectively, in *A. fuliginosus*, and are 1,419 and 773 bp in *B. bicolor*.

Both *A. fuliginosus* and *B. bicolor* have 22 tRNAs with sizes ranging from 62–75 bp, which are systematically embedded in each PCG, rRNA and AT-rich region. The total length of 22 tRNAs is 1,475 bp in *A. fuliginosus* and 1,475 bp in *B. bicolor*. Among the 22 tRNAs, 14 are encoded on the J strand and the remaining eight on the N strand, which is in accord with the other lepidopteran mitogenomes^28^. Most tRNA genes were folded into a cloverleaf secondary structure using MITOS, except for *trnS*^*(AGN)*^, which lacks the DHU arm both in *A. fuliginosus* and *B. bicolor* (Supplementary Material 2). In many insects, an ancestral status that lacks the DHU stem of *trnS*^*(AGN)*^ has been demonstrated^29^. In addition, the number of bases in the dihydrouridine loop ranges from 4 to 8 bp, which is not uniform because the DHU stem is highly variable^30^.

### Overlapping sequences, intergenic spacers and the control region

There are nine gene overlaps in *A. fuliginosus* and eight in *B. bicolor*, with sizes ranging from 1 to 8 bp. The maximum overlap of the two mitogenomes are located between *trnW* and *trnC* (Table 1). The length of the common overlap between *ATP6* and *ATP8*, which is widespread in hesperiid mitogenomes^18,31,32^, is 7 bp both in *A. fuliginosus* and *B. bicolor*.

The intergenic spacers of these two skippers are distributed among 15 regions, and their total lengths are 178 bp in *A. fuliginosus* and 135 bp in *B. bicolor*. Most of the intergenic spacers are not more than 20 bp. In these two mitogenomes, the longest, but not conserved, spacing sequence, whose position is similar to that in other hesperiid mitogenomes, is located between *trnQ* and *ND2*. This is consistent with this spacer probably arising in the process of gene rearrangements^23^.

**Figure 3:**
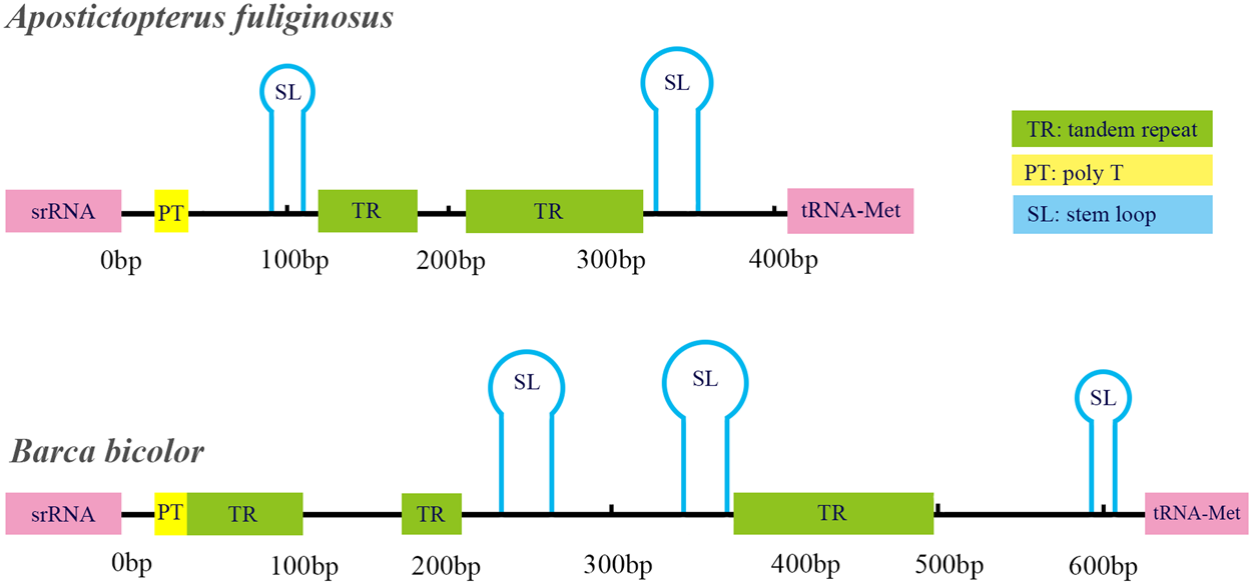
Predicted structural elements in the control region of *A. fuliginosus* and *B. bicolor*.

The control region is also called the AT-rich region because it is typically characterised by a high AT content. Moreover, the proportion of the AT content is as high as 94.6% in *A. fuliginosus* and 92% in *B. bicolor*. The control regions, the longest region of noncoding sequences that is located between the *srRNA* and *trnM*, are 407 bp and 614 bp in *A. fuliginosus* and *B. bicolor*, respectively. We found one dinucleotide repeat (TA)_55_ in *A. fuliginosus* and two dinucleotide repeats (TA)_36_ and (AT)_54_ in *B. bicolor*. Furthermore, we found a long tandem repeat of 30 bp (AAATAAAAAATTAAAATAATTATTTTAATT) in *A. fuliginosus* and a tandem repeat length of 18 bp (TAAAAAAATAATTATTTT) in *B. bicolor*. There was also a structure in the AT-rich region of both species with the poly-T stretch in a position close to the *srRNA*. Several microsatellite-like A/T sequences following the motif ATTTA in the control region were found in *A. fuliginosus* and *B. bicolor*, which were also discovered in the other skipper mitogenomes^33^. Moreover, our predicted results showed that there are two stem-loop structures in *A. fuliginosus* and three stem-loop structures in *B. bicolor* (Fig. 3). Many studies have shown that the motif ATAGA close to the 5ʹ-end of srRNA is greatly conserved^23,34^. This also exists in *A. fuliginosus* and *B. bicolor*.

### Phylogenetic analyses

Our datasets included 29 skippers for 14,715 nucleotides after removing ambiguous regions. Different strategies obtained almost the same results (see below); here, we present the results based on the PRT dataset as a basis for subsequent analyses. 16 best-fitting partitioning schemes (Supplementary Material S3) were determined by PartitionFinder with an initial subset of 63 possible partitions based on the PRT dataset.

**Figure 4:**
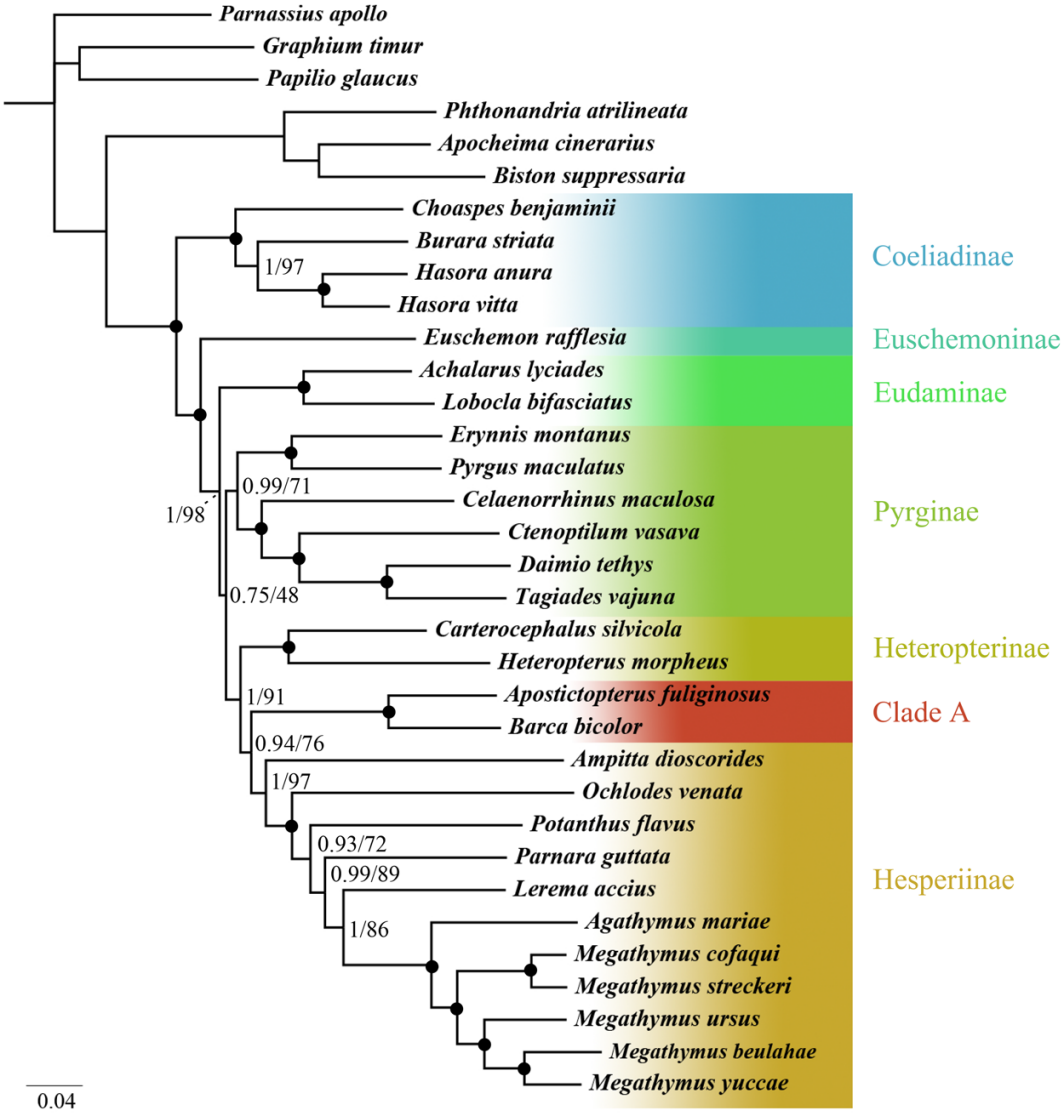
Phylogenetic tree using BI method based on PRT dataset. Numbers at node indicated posterior probabilities (PP) and bootstrap value (BS) based on ML analyses were also given. Dot on nodes means this branch: PP/BS = 1/100.

Similar topologies were inferred from phylogenetic analyses with MrBayes and IQ-TREE (Fig. 4). Six major clades were recovered: Coeliadinae, Euschemoninae, Eudaminae, Pyrginae, Heteropterinae, and Hesperiinae including subclade A, *A. fuliginosus* and *B. bicolor*, most of which agree with previous studies^1,3,6,10^. Coeliadinae is sister to the remaining subfamilies; the systematic positions of Euschemoninae and Eudaminae are confirmed, and Euschemoninae is the sister to all other skippers except Coeliadinae. Pyrginae, containing only four tribes (Erynnini, Pyrgini, Celaenorrhinini and Tagiadini), is recovered as monophyletic with weak support. Hesperiinae is obtained as monophyletic.

In the phylogenetic tree, *A. fuliginosus* and *B. bicolor* formed a strongly supported subclade (Clade A); this subclade branches after Heteropterinae and is followed by Hesperiinae with high support. Our results do not agree with placing them in the subfamily Heteropterinae^1,10^. We thus tentatively assign these two genera to the subfamily Hesperiinae. Previous studies have inferred a close relationship among Heteroptinae, Trapezitinae and Hesperiinae, but the sister relationships were uncertain^3,6^, and none of these studies sampled *Apostictopterus* and *Barca*. In this study, we were unable to include Trapezitinae to test for close relationships with Hesperiinae along with *Apostictopterus* and *Barca*, as no mitogenome is yet available. Hence, more samples in Trapezitinae are needed to confirm this hypothesis and clarify their systematic positions.

The phylogenetic analyses based on four datasets (PRT, PCGC, PCGD and PCGR) using two methods revealed very similar topologies except for the phylogenetic position of Eudaminae and Pyrginae. In the BI and ML analyses from different datasets, the topologies were largely congruent except for three strategies with little discrepancy. As many studies have concluded, the mitogenome can provide robust and stable phylogenetic analyses. The result from the PCGR dataset showed that Eudaminae branched after Euschemoninae in the BI analyses. In the ML analyses, however, the topologies based on the PCGC and PCGD datasets revealed that Eudaminae nested within Pyrginae (Supplementary Material S4), suggesting that Pyrginae is polyphyletic. Above all, the monophyly of Pyrginae and Eudaminae remains unresolved in our analyses, and more evidence is needed to address this issue.

## Materials and Methods

### Sample collection and DNA extraction

The adult specimen of *A. fuliginosus* was collected in Linzhi, Tibet Autonomous Region, China. The adult *B. bicolor* specimen was obtained in Weixi Lisu Autonomous County, Yunnan Province, China. Two or three legs from a single specimen were used to extract the genomic DNA using the HiPure Insect DNA Kit (Magen, China) following the manufacturer’s instructions.

### Primers, PCR, and cloning

For amplification, the complete mitogenomes were divided into 27 overlapping fragments. The primers were mainly taken from Kim *et al*.^23^ except for SF2, SF10, SF18, SF22 and SF27, which are newly designed (Supplementary Material S5). Due to the instability of the AT-rich region, we cloned this fragment after amplification and subsequent sequencing. For cloning, we referred to Fan *et al*.^35^.

We amplified all of the mitogenome but AT-rich regions using SuperMix (Transgene, China) via the following protocol: initial denaturation for 2 min at 94 °C, followed by 35 cycles of denaturation for 30 s at 94 °C, annealing for 45 s at 40–50 °C, and extension for 1 min at 72 °C, and a final extension step at 72 °C for 10 min. For the AT-rich region, we used KOD high-fidelity thermostable DNA polymerase (Takara, Japan) to improve the accuracy of the amplification and employed the following PCR conditions: initial denaturation of 2 min at 94 °C, followed by 35 cycles of 10 s at 98 °C, annealing for 45 s at 42 °C, and extension for 1 min at 68 °C, and a final extension at 72 °C for 10 min.

### Sequence analysis and annotation

We assembled and proof-read the sequences using the software Geneious v7.1.4^36^. PCGs were identified by finding the ORFs on the NCBI website (https://www.ncbi.nlm.nih.gov/orffinder/) with the invertebrate mitochondrial genetic codes. The tRNAs and rRNAs were identified using the MITOS Web Server (http://mitos.bioinf.uni-leipzig.de/index.py)^37^. Moreover, to confirm the accuracy of the boundaries of different genes, 37 genes were aligned using ClustalW in MEGA v7.0.2^38^ and manual inspection. The nucleotide composition statistics and relative synonymous codon usage (RSCU) were calculated using MEGA v7.0.2. The AT skew and GC skew^39^ values used for measuring the deviation of the base were calculated by the following formulas: AT skew = (A − T)/(A + T); GC skew = (G − C)/(G + C). The circular maps were drawn by CGView Server (http://stothard.afns.ualberta.ca/cgview_server/)^40^. The tandem repeats of the control region were identified with the Tandem Repeats Finder on-line server (http://tandem.bu.edu/trf/trf.html)^15^. Stem loop structures of the AT-rich region were predicted by DNAMAN. The two complete mitogenomes were deposited in GenBank with accession numbers MH985707 and MH985708.

### Phylogenetic analysis

We downloaded 33 available lepidopteran mitogenomes from GenBank, including 27 Hesperiidae, three Papilionidae and three Geometridae. The species used in this study are listed in Table 3. Each of the 13 PCGs was aligned individually using the software MAFFT V7.313^41^ with the G-INS-i strategy. Each of the two rRNAs was aligned separately using the Q-INS-i strategy through the MAFFT V7 online alignment server (https://mafft.cbrc.jp/alignment/server/)^42^. We removed gaps and ambiguous sites from the 13 PCGs by using the Gblocks V0.91^43^ online server (http://molevol.cmima.csic.es/castresana/Gblocks_server.html) with default settings.

**Table 3:** List of butterfly species analyzed in this paper with their respective GenBank accession numbers.

Species	Family	Size	GBAN*
*Achalarus lyciades*	Hesperiidae	15,612 bp	NC_030602
*Agathymus mariae*	Hesperiidae	15,342 bp	KY630504
*Ampittia dioscorides*	Hesperiidae	15,313 bp	KM102732
*Apostictopterus fuliginosus*	Hesperiidae	15,417 bp	MH985707
*Barca bicolor*	Hesperiidae	15,574 bp	MH985708
*Burara striata*	Hesperiidae	15,327 bp	NC_034676
*Carterocephalus silvicola*	Hesperiidae	15,765 bp	NC_024646
*Celaenorrhinus maculosa*	Hesperiidae	15,282 bp	NC_022853
*Choaspes benjaminii*	Hesperiidae	15,300 bp	NC_024647
*Ctenoptilum vasava*	Hesperiidae	15,468 bp	NC_016704
*Daimio tethys*	Hesperiidae	15,350 bp	NC_024648
*Erynnis montanus*	Hesperiidae	15,530 bp	NC_021427
*Euschemon rafflesia*	Hesperiidae	15,447 bp	NC_034231
*Hasora anura*	Hesperiidae	15,290 bp	NC_027263
*Hasora vitta*	Hesperiidae	15,282 bp	NC_027170
*Heteropterus morpheus*	Hesperiidae	15,769 bp	NC_028506
*Lerema accius*	Hesperiidae	15,338 bp	NC_029826
*Lobocla bifasciata*	Hesperiidae	15,366 bp	NC_024649
*Megathymus beulahae*	Hesperiidae	15,412 bp	KY630505
*Megathymus cofaqui*	Hesperiidae	15,421 bp	KY630503
*Megathymus streckeri*	Hesperiidae	15,507 bp	KY630501
*Megathymus ursus*	Hesperiidae	15,396 bp	KY630502
*Megathymus yuccae*	Hesperiidae	15,477 bp	KY630500
*Ochlodes venata*	Hesperiidae	15,622 bp	NC_018048
*Parnara guttatus*	Hesperiidae	15,441 bp	NC_029136
*Potanthus flavus*	Hesperiidae	15,267 bp	NC_024650
*Pyrgus maculatus*	Hesperiidae	15,346 bp	NC_030192
*Tagiades vajuna*	Hesperiidae	15,359 bp	KX865091
*Apocheima cinerarium*	Geometridae	15,722 bp	NC_024824
*Biston suppressaria*	Geometridae	15,628 bp	NC_027111
*Phthonandria atrilineata*	Geometridae	15,499 bp	NC_010522
*Graphium timur*	Papilionidae	15,226 bp	NC_024098
*Papilio glaucus*	Papilionidae	15,306 bp	NC_027252
*Parnassius apollo*	Papilionidae	15,404 bp	NC_024727

*GenBank accession number.

To compare the phylogenetic signal information of the different dataset combinations, four datasets were used: 1) PCGD: the 13 complete PCGs with the 3^rd^ codon removed; 2) PCGC: the 13 complete PCGs; 3) PRT: the 13 complete PCGs, two rRNAs and 22 tRNAs; and 4) PCGR: two rRNAs and 13 PCGs with the 3^rd^ codon removed. We employed PartitionFinder V2.1.1^44^ to identify the best partitioning strategies under the Bayesian information criterion (BIC). Maximum likelihood (ML) analyses were performed on the IQ-TREE web online server (http://iqtree.cibiv.univie.ac.at/)^45^ with 1000 ultrafast bootstraps (UFBS) to estimate the branch support. The best-fit models produced by ModelFinder^46^ implemented in IQ-tree. The Bayesian inference (BI) analyses were performed using MrBayes V3.2.6 on the CIPRES Science Gateway 3.3^47^. We used reversible-jump MCMC to allow sampling across all substitution rate models instead of specifying one substitution model, as suggested by PartitionFinder in BI analysis. Four Markov chains (one cold and three heated chains) were run simultaneously for 1 × 10^7^ generations with sampling every 1,000 generations. We examined the average standard change of the split frequencies in Tracer V1.7^48^ to determine the values falling below 0.01. We discarded the first 25% of the sampled trees as burn-in. The remaining trees were then used to calculate the posterior probabilities (PP) under the majority rule consensus.

## Electronic supplementary material

Supplementary information
