# Supplementary material for: The complete mitochondrial genomes of two skipper genera (Lepidoptera: Hesperiidae) and their associated phylogenetic analysis
Source: Sci Rep. 2018 Oct 25;8:15762. doi: 10.1038/s41598-018-34107-1 (PMC6202373; doi:10.1038/s41598-018-34107-1)
Supplement: Supplementary file 1 — Supplementary information [file 41598_2018_34107_MOESM1_ESM.pdf]

The complete mitochondrial genomes of two skipper genera (Lepidoptera: Hesperiidae) and their associated phylogenetic analysis

Yuke Han[1, *], Zhenfu Huang[1, *], Jing Tang[1], Hideyuki Chiba[2] & Xiaoling Fan[1]

[1]Department of Entomology, South China Agricultural University, College of Agriculture, Guangzhou, 510642, China.

[2]B. P. Bishop Museum, Honolulu, Hawaii, 96817-0916, U.S.A.

[*]These authors contributed equally to this work.

Correspondence and requests for materials should be addressed to X. F. (email: fanxiaol66@scau.edu.cn)

Supplementary Material S1. Composition and skewness of *Aposticopterus fuliginosus* and *Barca bicolor.*

Supplementary Material S2. The secondary structures of tRNA of both two species.

Supplementary Material S3. Best-fit partition schemes were selected by PartitionFinder and the corresponding best-fit model were selected by ModelFinder.

Supplementary Material S4. The trees based on different datasets and methods.

Supplementary Material S5. List of primers used to amplify and sequence two mitochondrial genomes.

Supplementary Material S1. Composition and skewness of *Aposticopterus fuliginosus* and *Barca bicolor.*

		T (bp)	A (bp)	C (bp)	G (bp)	Total Size (bp)	AT skew	GC skew
Aposticopterus fuliginosus	tRNA	609	609	147	110	1475	0	-0.144
	%	41.30	41.00	10.00	8.00			
	rRNA	971	900	217	101	2189	-0.038	-0.365
	%	44.4	41.1	9.9	4.6			
	AT-region	203	182	15	7	407	-0.055	-0.364
	%	49.9	44.7	3.7	1.7			
	PCGs	5088	3736	1169	1201	11194	-0.153	0.013
	%	45.5	33.4	10.4	10.7			
	Whole genome	6259	6188	1822	1148	15417	-0.006	-0.227
	%	40.6	40.1	11.8	7.4			
Barca bicolor	tRNA	597	592	158	117	1464	-0.004	-0.15
	%	40.80	40.40	10.80	8.00			
	rRNA	921	926	239	106	2192	0.003	-0.386
	%	42.2	10.9	4.8	42			
	AT-region	284	281	33	16	614	-0.005	-0.347
	%	46.3	45.8	5.4	2.6			
	PCGs	4997	3662	1262	1275	11196	-0.154	0.005
	%	44.6	32.7	11.3	11.4			
	Whole genome	6137	6227	2005	1205	15574	0.007	-0.249
	%	39.4	40	12.9	7.7			

Supplementary Material S2. The secondary structures of tRNA of both two species.

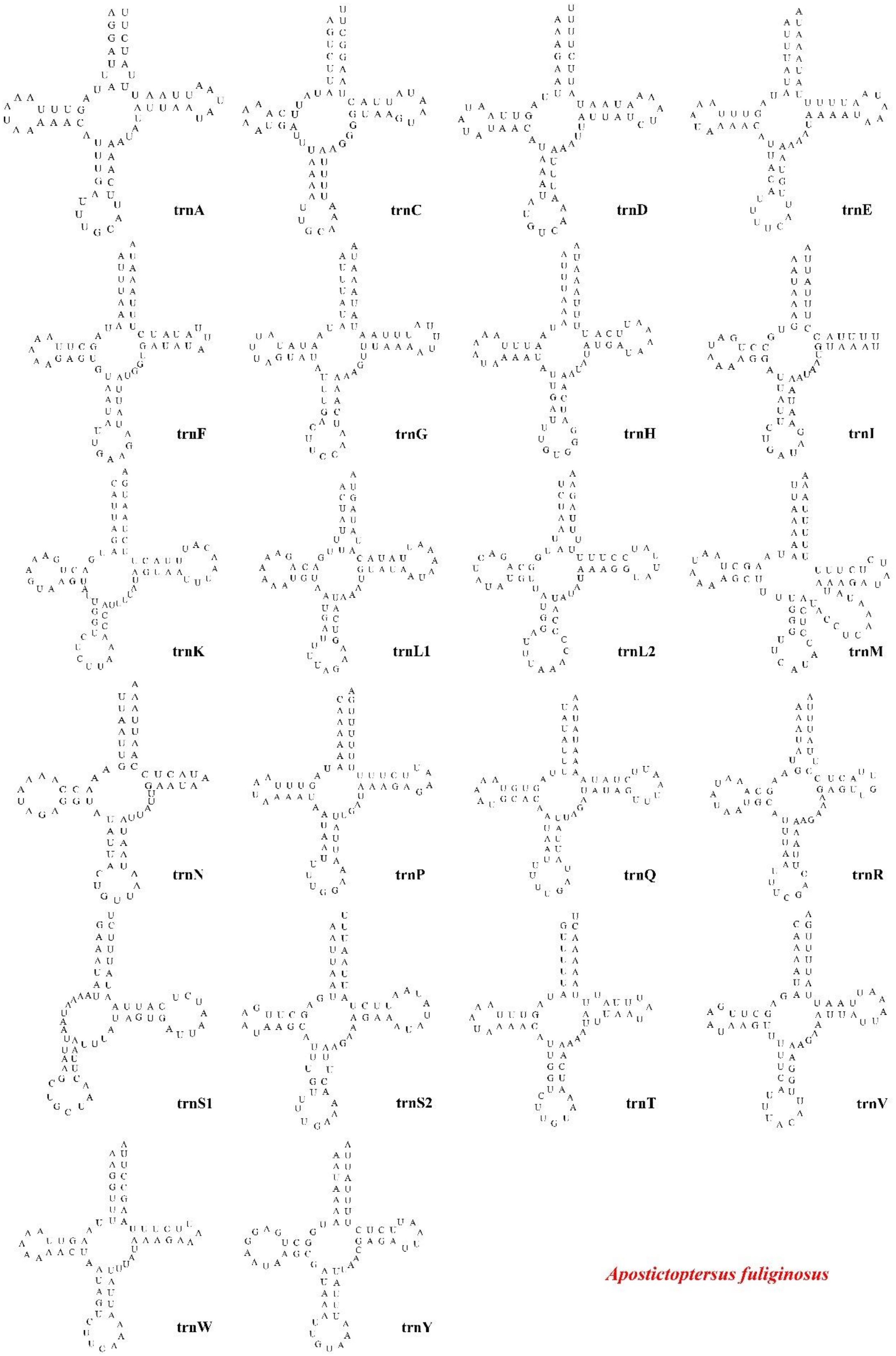

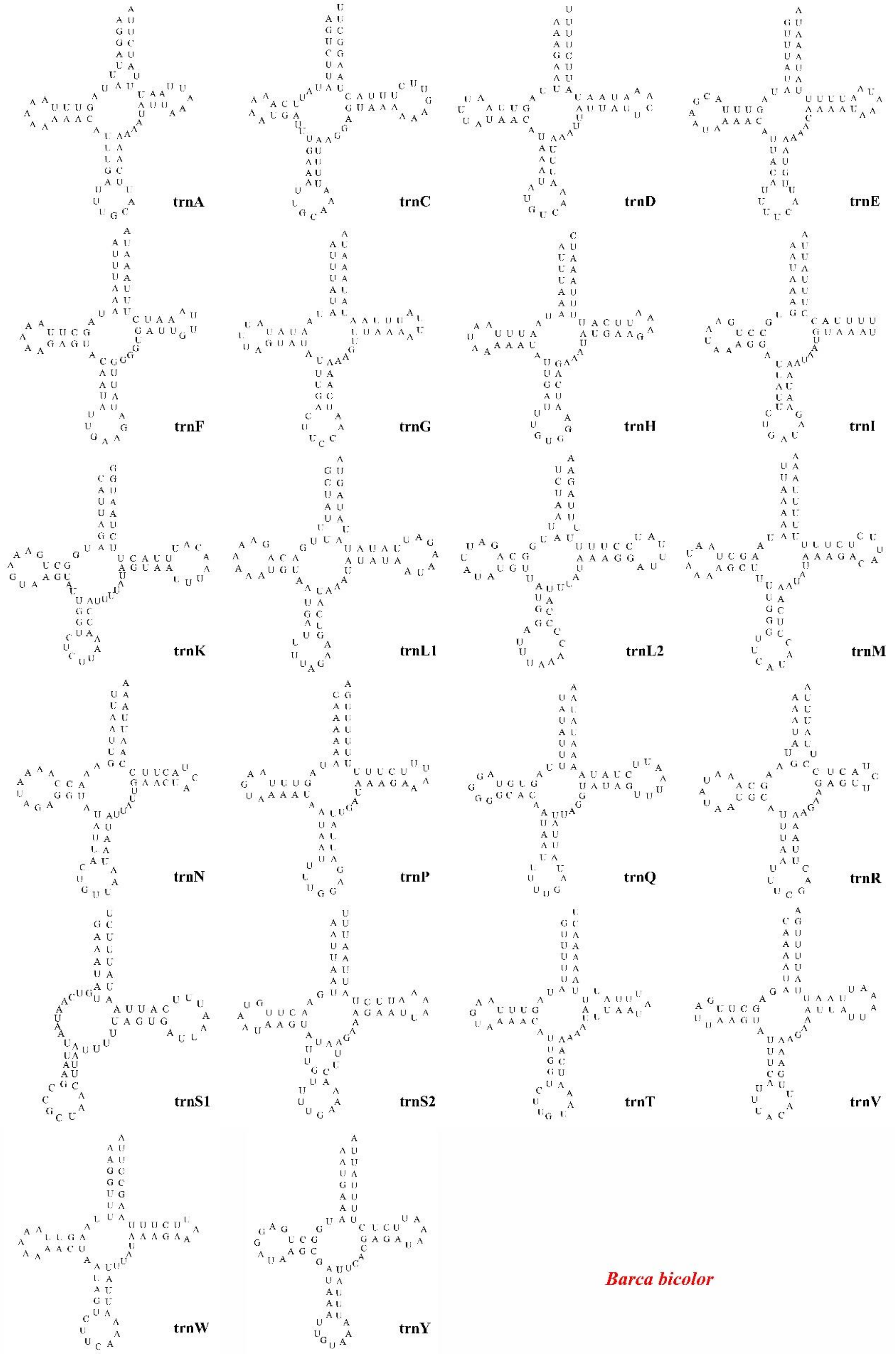
trnA
trnC
trnD
trnE
trnF
trnG
trnH
trnI
trnK
trnL1
trnL2
trnM
trnN
trnP
trnQ
trnR
trnS1
trnS2
trnT
trnV
trnW
trnY
Barca bicolor

Supplementary Material S3. Best-fit partition schemes were selected by PartitionFinder and the corresponding best-fit model were selected by ModelFinder.

ATP6_pos1 = 1-687\3;
ATP6_pos2 = 2-687\3;
ATP6_pos3 = 3-687\3;
ATP8_pos1 = 688-864\3;
ATP8_pos2 = 689-864\3;
ATP8_pos3 = 690-864\3;
COI_pos1 = 865-2415\3;
COI_pos2 = 866-2415\3;
COI_pos3 = 867-2415\3;
COII_pos1 = 2416-3112\3;
COII_pos2 = 2417-3112\3;
COII_pos3 = 2418-3112\3;
COIII_pos1 = 3113-3928\3;
COIII_pos2 = 3114-3928\3;
COIII_pos3 = 3115-3928\3;
CYTB_pos1 = 3929-5095\3;
CYTB_pos2 = 3930-5095\3;
CYTB_pos3 = 3931-5095\3;
ND1_pos1 = 5096-6052\3;
ND1_pos2 = 5097-6052\3;
ND1_pos3 = 5098-6052\3;
ND2_pos1 = 6053-7079\3;
ND2_pos2 = 6054-7079\3;
ND2_pos3 = 6055-7079\3;
ND3_pos1 = 7080-7436\3;
ND3_pos2 = 7081-7436\3;
ND3_pos3 = 7082-7436\3;
ND4_pos1 = 7437-8805\3;
ND4_pos2 = 7438-8805\3;
ND4_pos3 = 7439-8805\3;
ND4L_pos1 = 8806-9135\3;
ND4L_pos2 = 8807-9135\3;
ND4L_pos3 = 8808-9135\3;
ND5_pos1 = 9136-10908\3;
ND5_pos2 = 9137-10908\3;
ND5_pos3 = 9138-10908\3;
ND6_pos1 = 10909-11461\3;
ND6_pos2 = 10910-11461\3;
ND6_pos3 = 10911-11461\3;
lrRNA = 11462-12410;
srRNA = 12411-13001;

tRNA_Ala =13002-13087;
tRNA_Arg= 13088-13171;
tRNA_Asn =13172-13245;
tRNA_Asp= 13246-13321;
tRNA_Cys= 13322-13395;
tRNA_Gln= 13396-13464;
tRNA_Glu= 13465-13553;
tRNA_Gly= 13554-13625;
tRNA_His= 13626-13707;
tRNA_Ile= 13708-13779;
tRNA_Leu1= 13780-13871;
tRNA_Leu2= 13872-13940;
tRNA_Lys= 13941-14011;
tRNA_Met= 14012-14091;
tRNA_Phe= 14092-14162;
tRNA_Pro= 14163-14233;
tRNA_Ser= 14234-14317;
tRNA_Ser2= 14318-14402;
tRNA_Thr = 14403-14484;
tRNA_Trp = 14485-14556;
tRNA_Tyr = 14557-14628;
tRNA_Val = 14629-14715;

PCGD:

Partition:

charset Subset1 = 2416-3112\2 3929-5095\2 2417-3112\2 1-687\2 7080-7436\2 7081-7436\2 2-687\2 3930-5095\2 3114-3928\2 3113-3928\2;

charset Subset2 = 689-864\2 688-864\2 10910-11461\2 10909-11461\2;

charset Subset3 = 866-2415\2 865-2415\2;

charset Subset4 = 8807-9135\2 7437-8805\2 7438-8805\2 5096-6052\2 5097-6052\2 9137-10908\2 8806-9135\2 9136-10908\2;

charset Subset5 = 6053-7079\2 6054-7079\2;

ML charpartition mymodels =

GTR+F+I+G4: Subset1,
GTR+F+I+G4: Subset2,
GTR+F+I+G4: Subset3,
GTR+F+I+G4: Subset4,
TIM+F+I+G4: Subset5;

PCGC:

Partition:

charset Subset1 = 10909-11461\3 6053-7079\3 1-687\3 7080-7436\3;

charset Subset2 = 2417-3112\3 3114-3928\3 2-687\3 7081-7436\3 3930-5095\3;

charset Subset3 = 3-687\3 6055-7079\3;
charset Subset4 = 688-864\3 689-864\3;
charset Subset5 = 690-864\3 10911-11461\3 2418-3112\3 7082-7436\3 867-2415\3 3115-3928\3 3931-5095\3;
charset Subset6 = 865-2415\3;
charset Subset7 = 866-2415\3;
charset Subset8 = 2416-3112\3 3113-3928\3 3929-5095\3;
charset Subset9 = 7438-8805\3 8806-9135\3 5096-6052\3 9137-10908\3;
charset Subset10 = 8807-9135\3 5097-6052\3 9138-10908\3;
charset Subset11 = 5098-6052\3 8808-9135\3;
charset Subset12 = 10910-11461\3 6054-7079\3;
charset Subset13 = 7439-8805\3 7437-8805\3;
charset Subset14 = 9136-10908\3;

ML charpartition mymodels =

GTR+F+I+G4: Subset1,
TVM+F+I+G4: Subset2,
TN+F+G4: Subset3,
F81+F+I+G4: Subset4,
TIM+F+G4: Subset5,
TIM2+F+G4: Subset6,
F81+F+I+G4: Subset7,
GTR+F+I+G4: Subset8,
TIM+F+I+G4: Subset9,
GTR+F+I+G4: Subset10,
TPM2+F+I+G4: Subset11,
HKY+F+I+G4: Subset12,
GTR+F+I+G4: Subset13,
TPM2u+F+G4: Subset14;

PCGR:

Partition:

charset Subset1 = 2416-3112\2 2417-3112\2 3929-5095\2 1-687\2 7080-7436\2 7081-7436\2 2-687\2 3930-5095\2 3114-3928\2 3113-3928\2;
charset Subset2 = 689-864\2 688-864\2 10909-11461\2 10910-11461\2;
charset Subset3 = 866-2415\2 865-2415\2;
charset Subset4 = 8807-9135\2 7437-8805\2 7438-8805\2 5096-6052\2 5097-6052\2 9137-10908\2 8806-9135\2 9136-10908\2;
charset Subset5 = 6053-7079\2 6054-7079\2;
charset Subset6 = 12411-13001 11462-12410;

ML charpartition mymodels =

GTR+F+I+G4: Subset1,
GTR+F+I+G4: Subset2,
GTR+F+I+G4: Subset3,
GTR+F+I+G4: Subset4,

TIM+F+I+G4: Subset5,
GTR+F+I+G4: Subset6;

PRT:

Partition:

charset Subset1 = 14092-14162 13626-13707 10909-11461\3 6053-7079\3 13780-13871 1-687\3 7080-7436\3;
charset Subset2 = 13941-14011 13872-13940 2-687\3 2417-3112\3 3114-3928\3 3930-5095\3;
charset Subset3 = 3-687\3 6055-7079\3;
charset Subset4 = 688-864\3 689-864\3;
charset Subset5 = 690-864\3 10911-11461\3 2418-3112\3 3931-5095\3 867-2415\3 3115-3928\3 7082-7436\3;
charset Subset6 = 865-2415\3;
charset Subset7 = 866-2415\3;
charset Subset8 = 13396-13464 2416-3112\3;
charset Subset9 = 3113-3928\3 3929-5095\3 13322-13395 14485-14556 14012-14091;
charset Subset10 = 7438-8805\3 13708-13779 8806-9135\3 5096-6052\3 9137-10908\3;
charset Subset11 = 8807-9135\3 9138-10908\3 5097-6052\3;
charset Subset12 = 5098-6052\3;
charset Subset13 = 7081-7436\3 10910-11461\3 6054-7079\3;
charset Subset14 = 7439-8805\3 7437-8805\3;
charset Subset15 = 9136-10908\3 8808-9135\3;
charset Subset16 = 14163-14233 14557-14628 14629-14715 12411-13001 11462-12410 13246-13321 13465-13553 13088-13171 13172-13245 13554-13625 14318-14402 14234-14317 13002-13087 14403-14484;

ML charpartition mymodels =

GTR+F+I+G4: Subset1,
TVM+F+I+G4: Subset2,
TN+F+G4: Subset3,
F81+F+I+G4: Subset4,
TIM+F+G4: Subset5,
TIM2+F+G4: Subset6,
F81+F+I+G4: Subset7,
TN+F+I+G4: Subset8,
GTR+F+I+G4: Subset9,
TIM+F+I+G4: Subset10,
GTR+F+I+G4: Subset11,
TIM2+F+G4: Subset12,
HKY+F+I+G4: Subset13,
GTR+F+I+G4: Subset14,
TIM+F+I+G4: Subset15,
GTR+F+I+G4: Subset16;

Supplementary Material S4. The trees based on different datasets and methods.

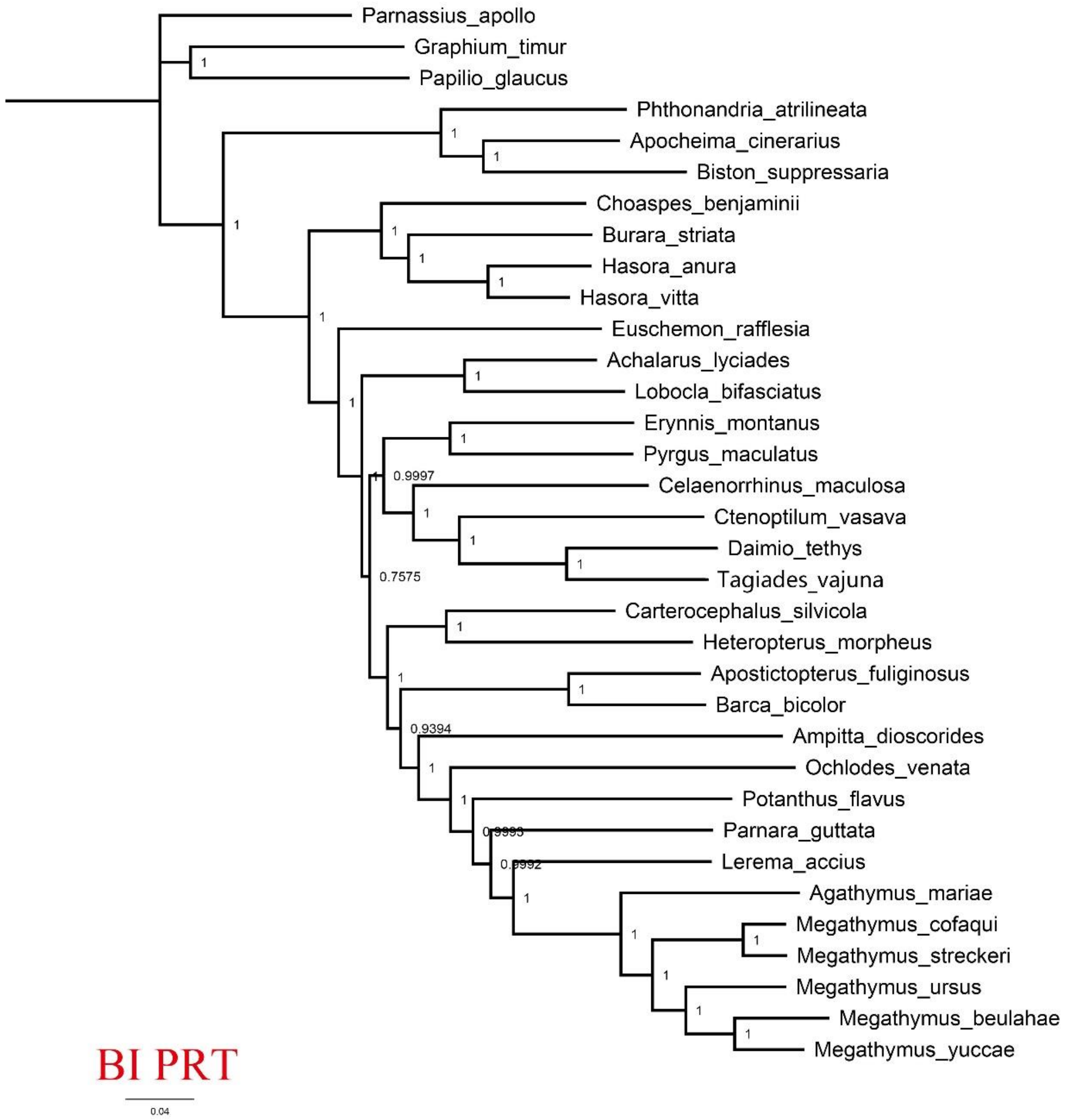

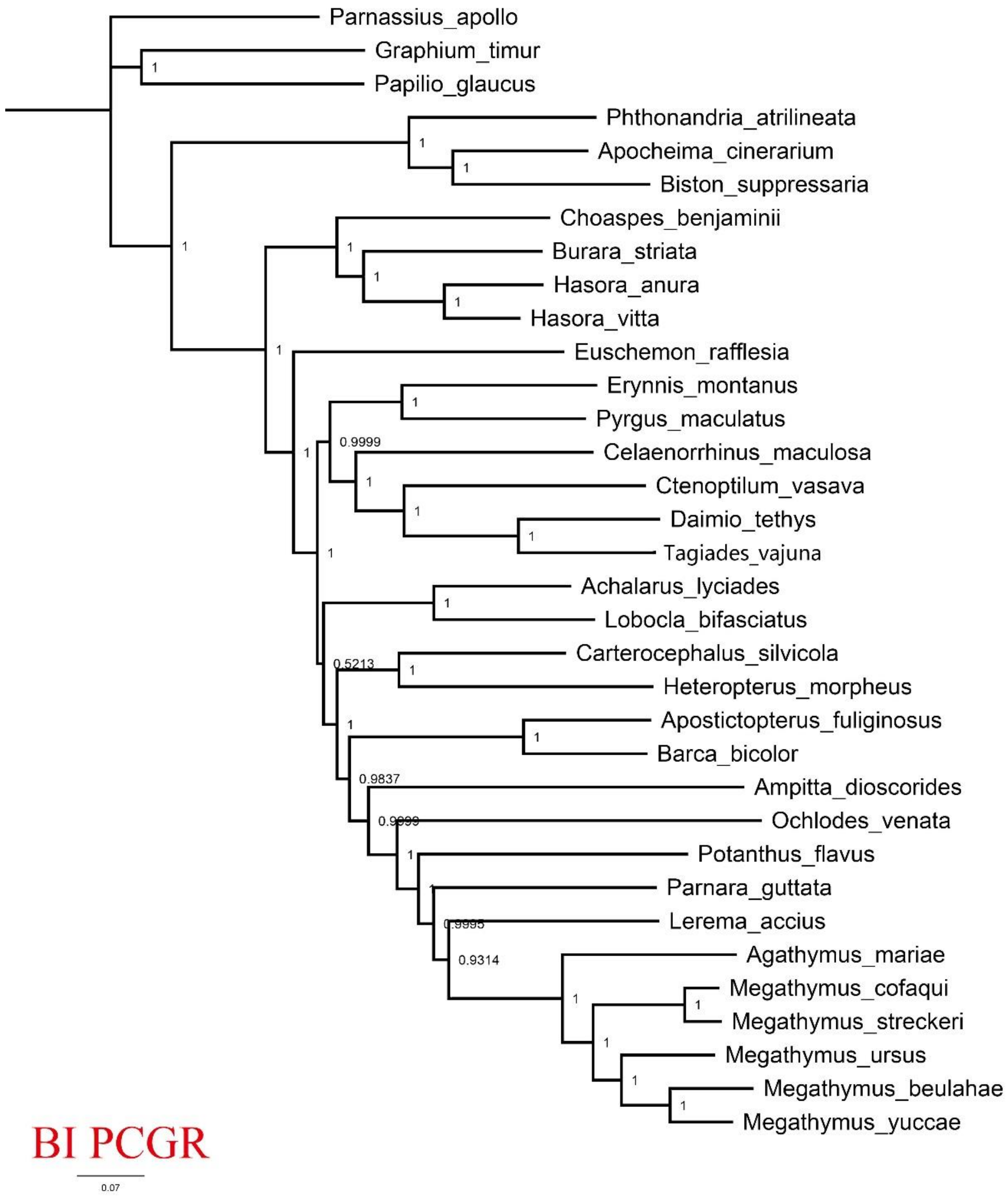
Parnassius_apollo
Graphium_timur
Papilio_glaucus
Phthonandria_atrilineata
Apocheima_cinerarium
Biston_suppressaria
Choaspes_benjaminii
Burara_striata
Hasora_anura
Hasora_vitta
Euschemon_rafflesia
Erynnis_montanus
Pyrgus_maculatus
Celaenorrhinus_maculosa
Ctenoptilum_vasava
Daimio_tethys
Tagiades_vajuna
Achalarus_lyciades
Lobocla_bifasciatus
Carterocephalus_silvicola
Heteropterus_morpheus
Apostictopterus_fuliginosus
Barca_bicolor
Ampitta_dioscorides
Ochlodes_venata
Potanthus_flavus
Parnara_guttata
Lerema_accius
Agathymus_mariae
Megathymus_cofaqui
Megathymus_streckeri
Megathymus_ursus
Megathymus_beulahae
Megathymus_yuccae
0.9999
0.5213
0.9837
0.9314
0.07
BI PCGR

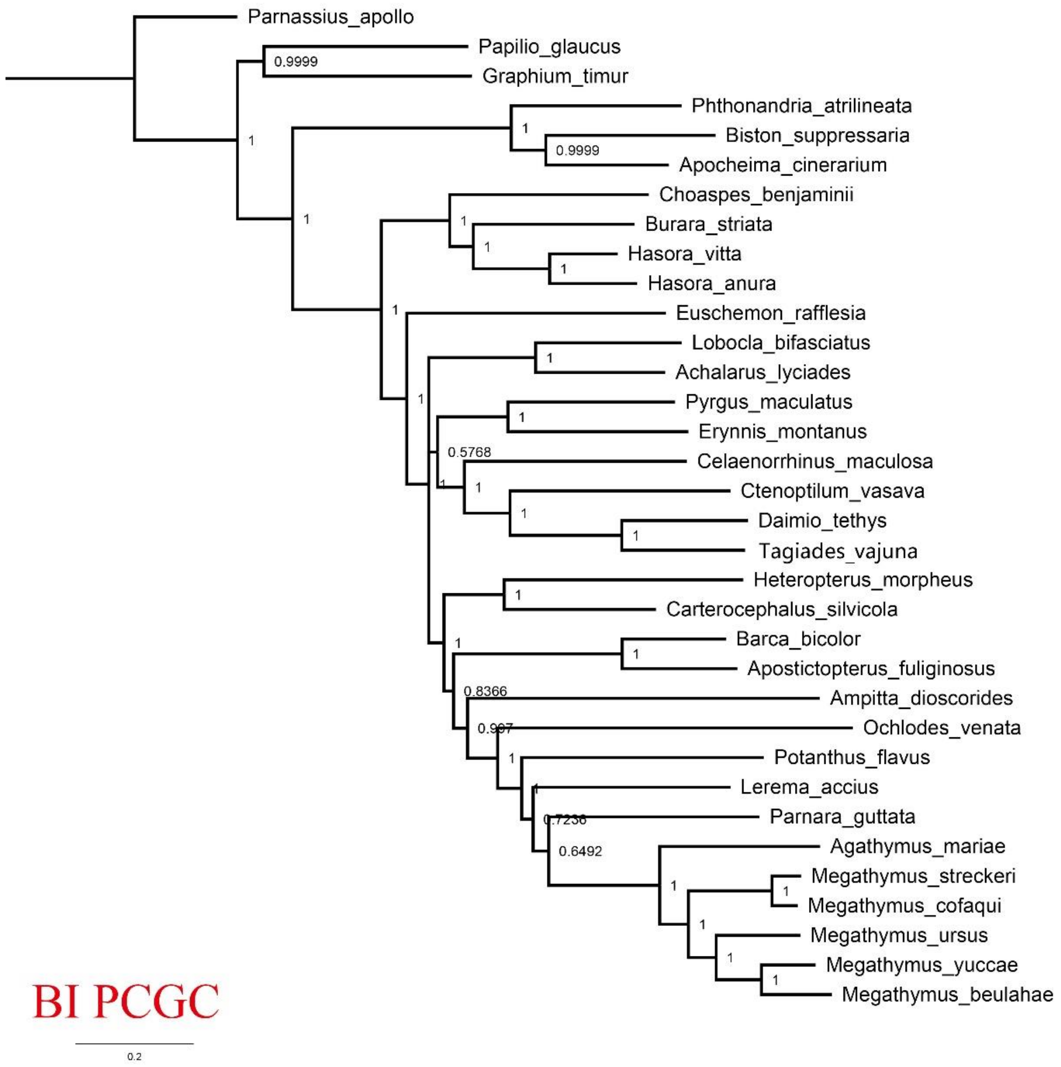
Parnassius_apollo
Papilio_glaucus
0.9999
Graphium_timur
Phthonandria_atrilineata
1
Biston_suppressaria
0.9999
Apocheima_cinerarium
Choaspes_benjaminii
Burara_striata
Hasora_vitta
Hasora_anura
Euschemon_rafflesia
Lobocla_bifasciatus
Achalarus_lyciades
Pyrgus_maculatus
Erynnis_montanus
0.5768
Celaenorrhinus_maculosa
Ctenoptilum_vasava
Daimio_tethys
Tagiades_vajuna
Heteropterus_morpheus
Carterocephalus_silvicola
Barca_bicolor
Apostictopterus_fuliginosus
0.8366
Ampitta_dioscorides
Ochlodes_venata
Potanthus_flavus
Lerema_accius
0.7236
Parnara_guttata
0.6492
Agathymus_mariae
Megathymus_streckeri
Megathymus_cofaqui
Megathymus_ursus
Megathymus_yuccae
Megathymus_beulahae
BI PCGC
0.2

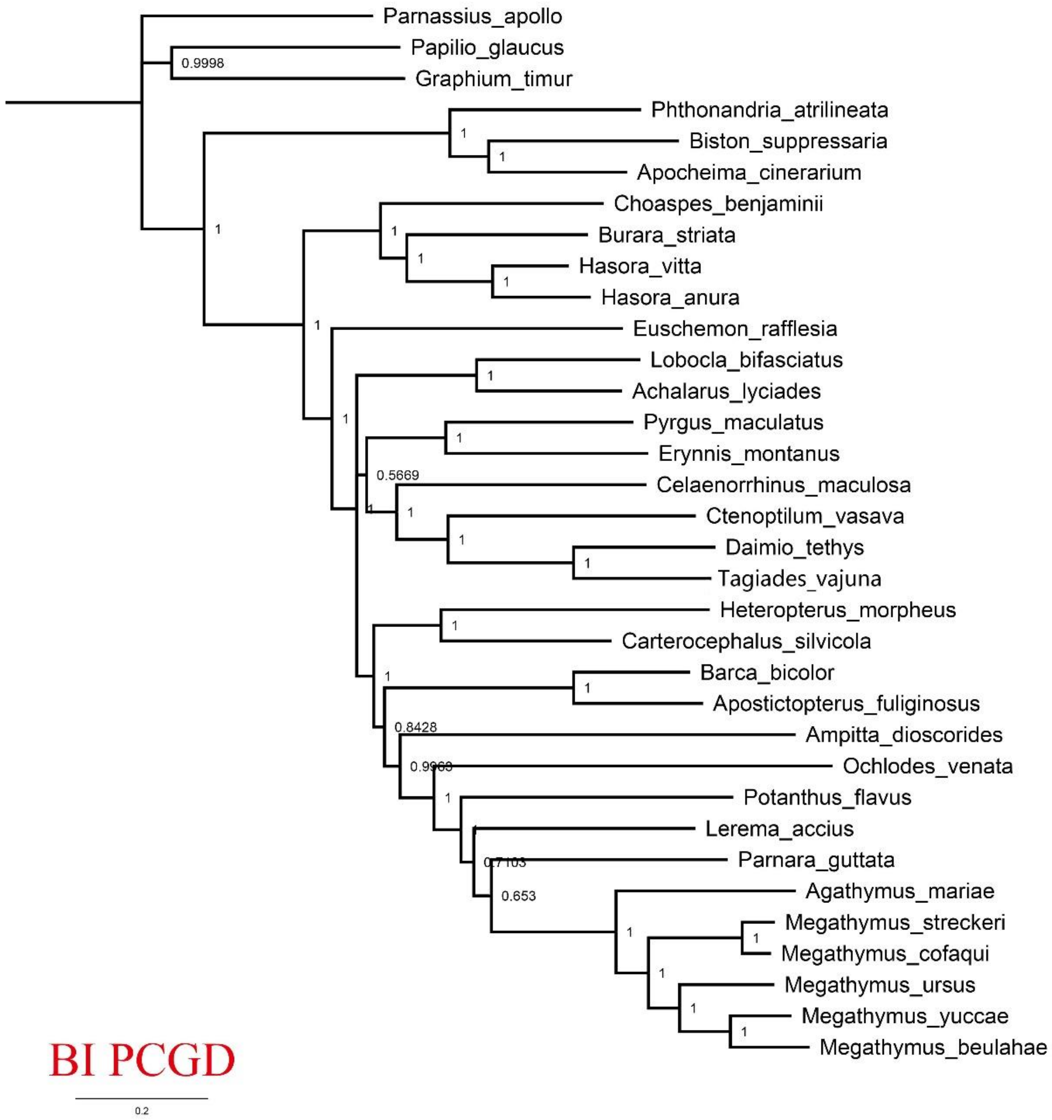
Parnassius_apollo
Papilio_glaucus
0.9998
Graphium_timur
Phthonandria_atrilineata
Biston_suppressaria
Apocheima_cinerarium
Choaspes_benjaminii
Burara_striata
Hasora_vitta
Hasora_anura
Euschemon_rafflesia
Lobocla_bifasciatus
Achalarus_lyciades
Pyrgus_maculatus
Erynnis_montanus
0.5669
Celaenorrhinus_maculosa
Ctenoptilum_vasava
Daimio_tethys
Tagiades_vajuna
Heteropterus_morpheus
Carterocephalus_silvicola
Barca_bicolor
Apostictopterus_fuliginosus
0.8428
Ampitta_dioscorides
Ochlodes_venata
Potanthus_flavus
Lerema_accius
Parnara_guttata
0.653
Agathymus_mariae
Megathymus_streckeri
Megathymus_cofaqui
Megathymus_ursus
Megathymus_yuccae
Megathymus_beulahae
BI PCGD
0.2

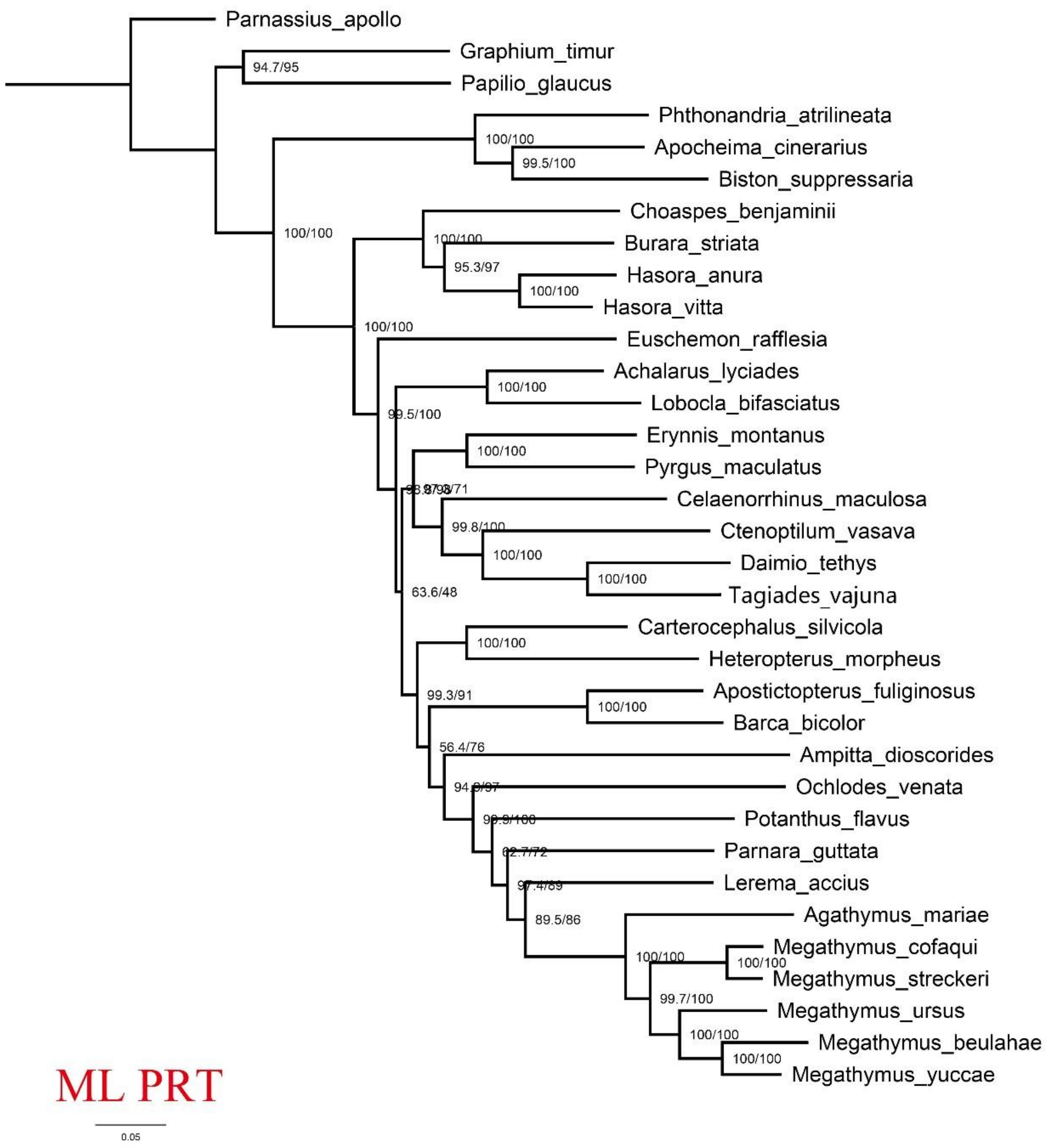
Parnassius_apollo
Graphium_timur
94.7/95
Papilio_glaucus
Phthonandria_atrilineata
100/100
Apocheima_cinerarius
99.5/100
Biston_suppressaria
100/100
Choaspes_benjaminii
100/100
Burara_striata
95.3/97
Hasora_anura
100/100
Hasora_vitta
100/100
Euschemon_rafflesia
Achalarus_lyciades
100/100
Lobocla_bifasciatus
99.5/100
Erynnis_montanus
100/100
Pyrgus_maculatus
Celaenorrhinus_maculosa
99.8/100
Ctenoptilum_vasava
100/100
Daimio_tethys
100/100
Tagiades_vajuna
63.6/48
Carterocephalus_silvicola
100/100
Heteropterus_morpheus
99.3/91
Apostictopterus_fuliginosus
100/100
Barca_bicolor
56.4/76
Ampitta_dioscorides
Ochlodes_venata
Potanthus_flavus
82.7/72
Parnara_guttata
Lerema_accius
89.5/86
Agathymus_mariae
100/100
Megathymus_cofaqui
100/100
Megathymus_streckeri
99.7/100
Megathymus_ursus
100/100
Megathymus_beulahae
100/100
Megathymus_yuccae
ML PRT
0.05

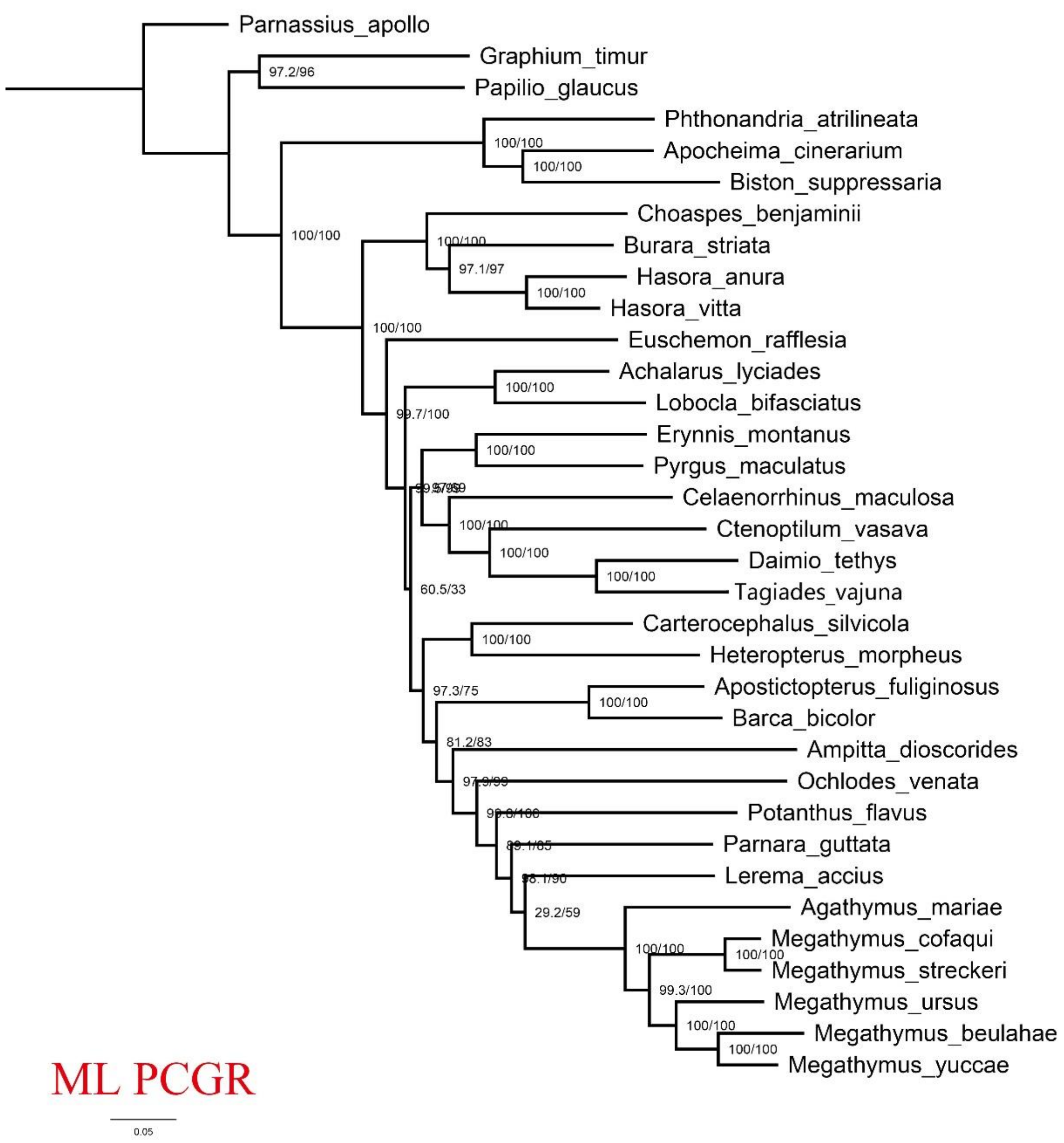
Parnassius_apollo
Graphium_timur
97.2/96
Papilio_glaucus
Phthonandria_atrilineata
100/100
Apocheima_cinerarium
100/100
Biston_suppressaria
100/100
Choaspes_benjaminii
Burara_striata
97.1/97
Hasora_anura
100/100
Hasora_vitta
100/100
Euschemon_rafflesia
Achalarus_lyciades
100/100
Lobocla_bifasciatus
99.7/100
Erynnis_montanus
100/100
Pyrgus_maculatus
Celaenorrhinus_maculosa
Ctenoptilum_vasava
100/100
Daimio_tethys
100/100
Tagiades_vajuna
60.5/33
Carterocephalus_silvicola
100/100
Heteropterus_morpheus
97.3/75
Apostictopterus_fuliginosus
100/100
Barca_bicolor
81.2/83
Ampitta_dioscorides
Ochlodes_venata
Potanthus_flavus
Parnara_guttata
Lerema_accius
29.2/59
Agathymus_mariae
100/100
Megathymus_cofaqui
100/100
Megathymus_streckeri
99.3/100
Megathymus_ursus
100/100
Megathymus_beulahae
100/100
Megathymus_yuccae
ML PCGR
0.05

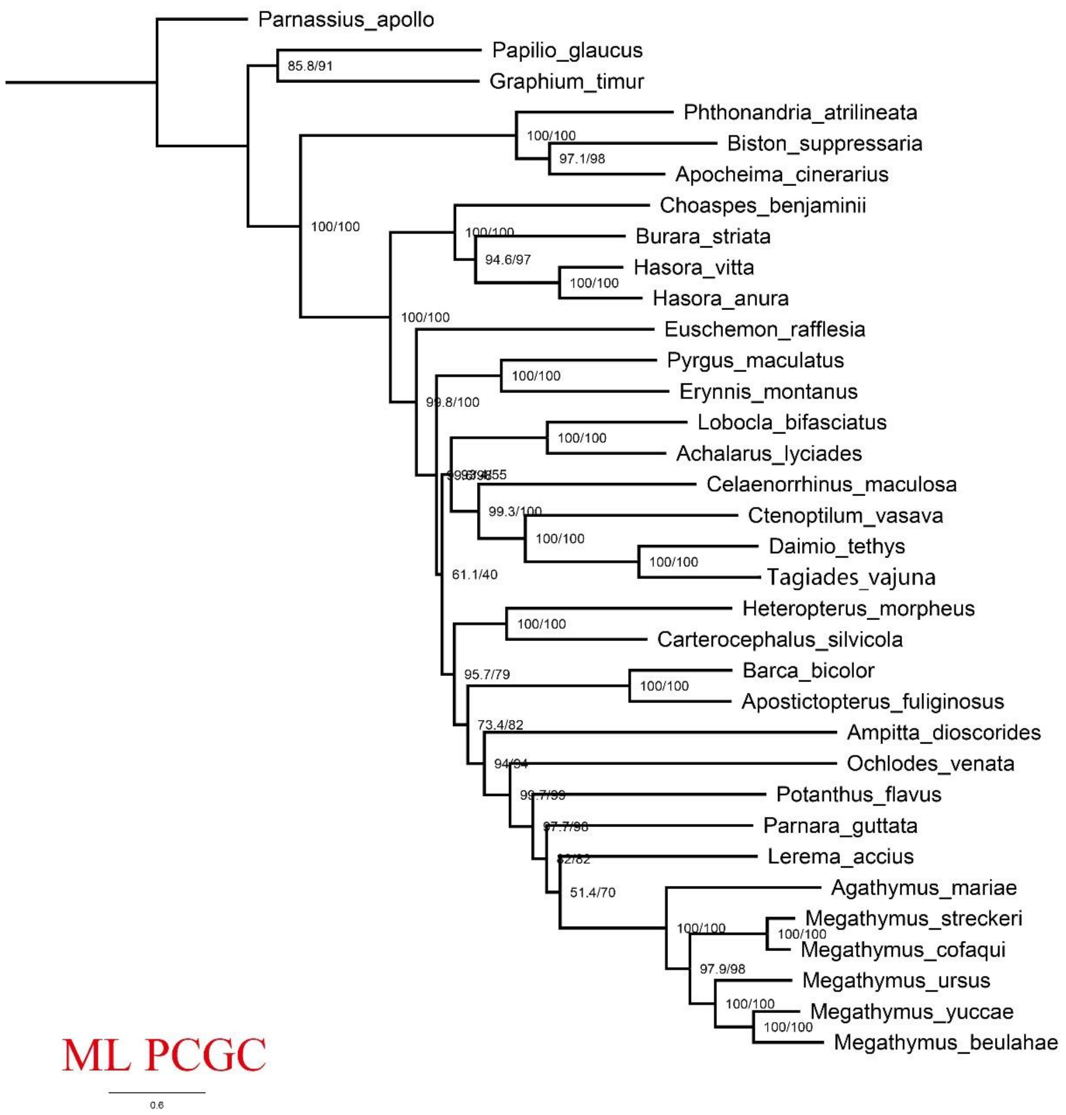
Parnassius_apollo
Papilio_glaucus
85.8/91
Graphium_timur
Phthonandria_atrilineata
100/100
Biston_suppressaria
97.1/98
Apocheima_cinerarius
100/100
Choaspes_benjaminii
100/100
Burara_striata
94.6/97
Hasora_vitta
100/100
Hasora_anura
100/100
Euschemon_rafflesia
Pyrgus_maculatus
100/100
99.8/100
Erynnis_montanus
Lobocla_bifasciatus
100/100
Achalarus_lyciades
Celaenorrhinus_maculosa
99.3/100
Ctenoptilum_vasava
100/100
Daimio_tethys
100/100
Tagiades_vajuna
61.1/40
Heteropterus_morpheus
100/100
Carterocephalus_silvicola
95.7/79
Barca_bicolor
100/100
Apostictopterus_fuliginosus
73.4/82
Ampitta_dioscorides
94/94
Ochlodes_venata
Potanthus_flavus
Parnara_guttata
Lerema_accius
51.4/70
Agathymus_mariae
100/100
Megathymus_streckeri
100/100
Megathymus_cofaqui
97.9/98
Megathymus_ursus
100/100
Megathymus_yuccae
100/100
Megathymus_beulahae
ML PCGC
0.6

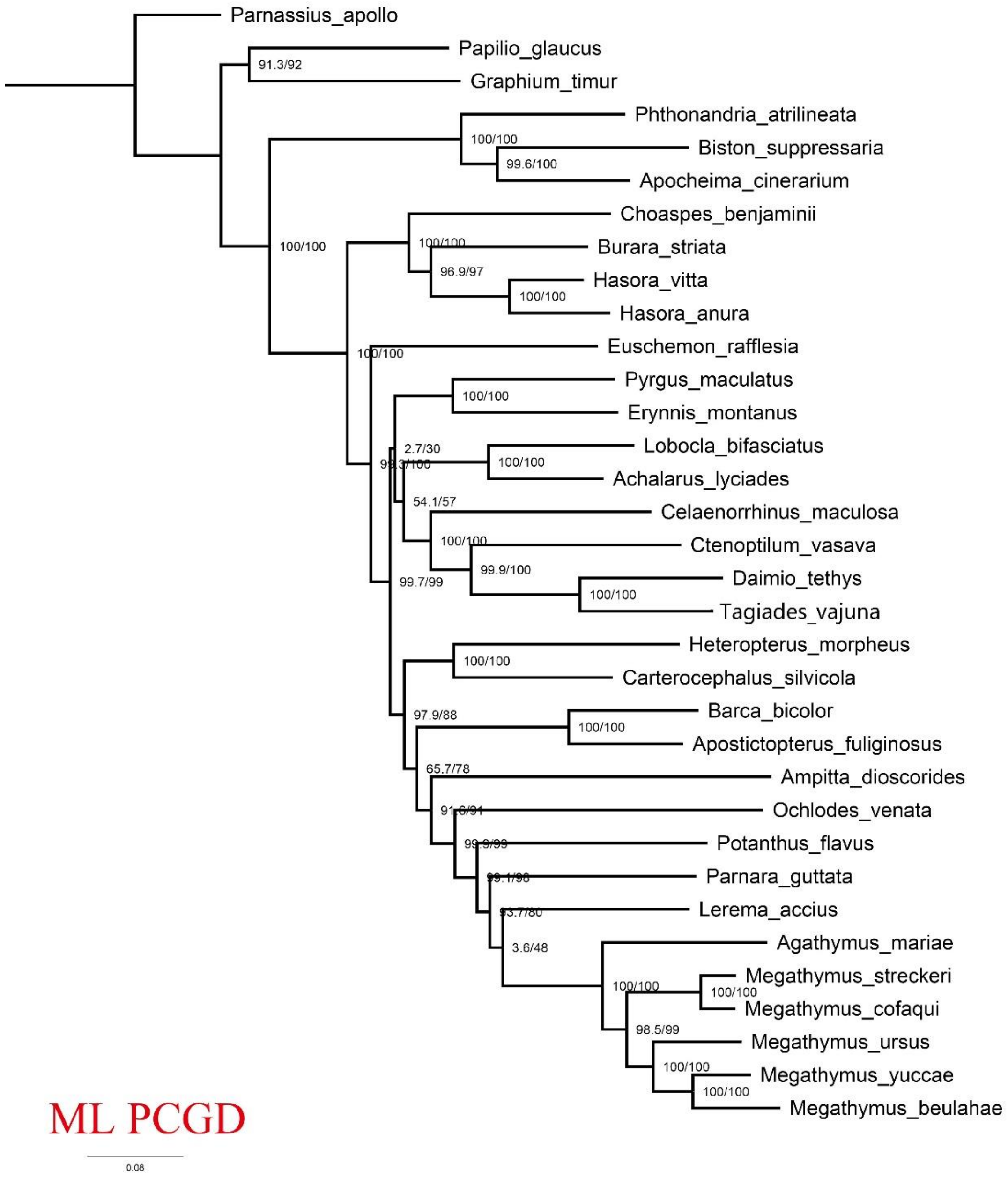
Parnassius_apollo
Papilio_glaucus
91.3/92
Graphium_timur
Phthonandria_atrilineata
100/100
Biston_suppressaria
99.6/100
Apocheima_cinerarium
Choaspes_benjaminii
100/100
100/100
Burara_striata
96.9/97
Hasora_vitta
100/100
Hasora_anura
Euschemon_rafflesia
100/100
Pyrgus_maculatus
100/100
Erynnis_montanus
2.7/30
Lobocla_bifasciatus
100/100
Achalarus_lyciades
54.1/57
Celaenorrhinus_maculosa
100/100
Ctenoptilum_vasava
99.9/100
99.7/99
Daimio_tethys
100/100
Tagiades_vajuna
Heteropterus_morpheus
100/100
Carterocephalus_silvicola
97.9/88
Barca_bicolor
100/100
Apostictopterus_fuliginosus
65.7/78
Ampitta_dioscorides
91.6/91
Ochlodes_venata
Potanthus_flavus
Parnara_guttata
Lerema_accius
3.6/48
Agathymus_mariae
100/100
Megathymus_streckeri
100/100
Megathymus_cofaqui
98.5/99
Megathymus_ursus
100/100
Megathymus_yuccae
100/100
Megathymus_beulahae
ML PCGD
0.08

Supplementary Material S5. List of primers used to amplify and sequence two mitochondrial genomes.

Fragment name	Gene	Direction[a]	Sequence (5'- 3')
SF1	*tRNA*^{Met}	F	AAGCTTTTGGGYTCATACC
	ND2	R	CAWCCTAAATTATTAATWGAWGA
SF2*	*ND2*	F	TCTTCWATAATAATRAAAATAGG
	tRNA^{Tyr}	R	GCGATAAATTGTAAATTTAT
SF3	*tRNA*^{Trp}	F	AATCTTCAAAATTATTTATAAAG
	COI	R	CTTCAGGATGACCAAAAAATC
SF4	*COI*	F	GGTATTTCATCAATTTTAGG
	COI	R	GTCGAGGTATTCCTGCTA
SF5	*COI*	F	ACWGTAGGAGGATTAACAGG
	COII	R	GTTCAAATTAATTCAATTATTTG
SF6	*COII*	F	TAGAAATGGCAACWTGATC
	tRNA^{Lys}	R	CTTGCTTTCAGTCATCTAAT
SF7	*COII*	F	CAGGTCGWTTAAATCAAAC
	ATP6	R	GTTCCTTGDGGAATTATATG
SF8	*ATP6*	F	TTATTTTCAATTTTTGATCC
	COIII	R	CTCGTCATCATTGATATAT
SF9	*COIII*	F	GTWGATTATAGHCCWTGACC
	tRNA^{Gly}	R	GATTGGAAGTCAAATATACT
SF10*	*COIII*	F	AGCTTATGAATATTAYGAAGC
	tRNA^{Asn}	R	CAATTTTATCATTAACAGTGA
SF11	*ND3*	F	TAGAAATTGCATTAATTTTHCC
	ND5	R	CCTTATATAATTTATTTACC
SF12	*ND5*	F	AWAHTTCTCTTCAACCYAWATC
	ND5	R	GCTTTATCWACTTTAAGWCA
SF13	*ND5*	F	TCYTTWGAATAAAAYCCAG
	ND5	R	GATGGDTTAGGDTTAGTTTCTT
SF14	*ND5*	F	AAAAAATATAATTTCAWCTHCC
	ND4	R	CATTGATTWCCTTTAAATAT
SF15	*tRNA*^{His}	F	ATATTTTTGAYHCCACAAATC
	ND4	R	CAGGTTCAATAATTTTAGC
SF16	*ND4*	F	TTATAATACCHCCAATWAC
	ND4	R	GGTTTAATTTTATTAAGAATTTG
SF17	*ND4*	F	ATATTAAAGTAGGAATTAAWC
	tRNA^{Pro}	R	TAATTTTGGAGATTATWGAT
SF18*	*ND4L*	F	CCTAAAGCACCCTCACAWAC

	ND6	R	GTAATTTTTACWACWGMWAYTA
SF19	*ND6*	F	TNTCAAGAATTGCHTCWAATG
	Cytb	R	GATATTTGTCCYCAAGGTA
SF20	*Cytb*	F	TATHTHCATATTGGACGAGG
	Cytb	R	CCTTGDATTTTTTTATTAAADGT
SF21	*Cytb*	F	ACHCCHRTTCATATTCAACC
	ND1	R	GCTGAAACTAATCGAACTC
SF22*	*ND1*	F	ACCGRGGWARMGTMCCTCG
	lrRNA	R	CTGAGTTCAAACCGGTGTRA
SF23	*tRNA^{Leu}*	F	GAHTTCTAAAAYCATTAC
	lrRNA	R	GACTGTACAAAGGTAGCATAAT
SF24	*lrRNA*	F	CATCMAATCTTTCATACAAG
	lrRNA	R	GTATCTTGTGTATCAGAG
SF25	*lrRNA*	F	ATTATGCTACCTTTGTACAGTC
	tRNA^{Val}	R	GTATTTCATTTACATTGAAAAGA
SF26	*lrRNA*	F	CTCTGATACACAAGATAC
	srRNA	R	CCAGCAGTTGCGGTTAAAC
SF27*	*srRNA*	F	AATAGGTGATCTAATCCTAG
	tRNA^{Ile}	R	CTATCAGAATAATCCTTTWW

[a]F and R, forward and reverse direction of trsnacription.

*Primers are newly designed in this study.
